# Diffusion tensor imaging in anisotropic tissues: application of reduced gradient vector schemes in peripheral nerves

**DOI:** 10.1186/s41747-024-00444-2

**Published:** 2024-04-02

**Authors:** Olivia Foesleitner, Alba Sulaj, Volker Sturm, Moritz Kronlage, Fabian Preisner, Zoltan Kender, Martin Bendszus, Julia Szendroedi, Sabine Heiland, Daniel Schwarz

**Affiliations:** 1 Department of Neuroradiology, Heidelberg University Hospital, INF 400, 69120 Heidelberg, Germany; 2 Department of Internal Medicine I and Clinical Chemistry, Heidelberg University Hospital, INF 410, Heidelberg, Germany; 3 German Center of Diabetes Research (DZD), Neuherberg, Germany; 4 Joint Heidelberg-IDC Translational Diabetes Program, Helmholtz Center Munich, Neuherberg, Germany

**Keywords:** Diabetes mellitus (type 2), Diffusion tensor imaging, Healthy volunteers, Magnetic resonance imaging, Peripheral nerves

## Abstract

**Background:**

In contrast to the brain, fibers within peripheral nerves have distinct monodirectional structure questioning the necessity of complex multidirectional gradient vector schemes for DTI. This proof-of-concept study investigated the diagnostic utility of reduced gradient vector schemes in peripheral nerve DTI.

**Methods:**

Three-Tesla magnetic resonance neurography of the tibial nerve using 20-vector DTI (DTI_20_) was performed in 10 healthy volunteers, 12 patients with type 2 diabetes, and 12 age-matched healthy controls. From the full DTI_20_ dataset, three reduced datasets including only two or three vectors along the *x*- and/or *y*- and *z*-axes were built to calculate major parameters. The influence of nerve angulation and intraneural connective tissue was assessed. The area under the receiver operating characteristics curve (ROC-AUC) was used for analysis.

**Results:**

Simplified datasets achieved excellent diagnostic accuracy equal to DTI_20_ (ROC-AUC 0.847–0.868, *p* ≤ 0.005), but compared to DTI_20_, the reduced models yielded mostly lower absolute values of DTI scalars: median fractional anisotropy (FA) ≤ 0.12; apparent diffusion coefficient (ADC) ≤ 0.25; axial diffusivity ≤ 0.96, radial diffusivity ≤ 0.07). The precision of FA and ADC with the three-vector model was closest to DTI_20_. Intraneural connective tissue was negatively correlated with FA and ADC (*r* ≥ -0.49, *p* < 0.001). Small deviations of nerve angulation had little effect on FA accuracy.

**Conclusions:**

In peripheral nerves, bulk tissue DTI metrics can be approximated with only three predefined gradient vectors along the scanner’s main axes, yielding similar diagnostic accuracy as a 20-vector DTI, resulting in substantial scan time reduction.

**Relevance statement:**

DTI bulk tissue parameters of peripheral nerves can be calculated with only three predefined gradient vectors at similar diagnostic performance as a standard DTI but providing a substantial scan time reduction.

**Key points:**

• In peripheral nerves, DTI parameters can be approximated using only three gradient vectors.

• The simplified model achieves a similar diagnostic performance as a standard DTI.

• The simplified model allows for a significant acceleration of image acquisition.

• This can help to introduce multi-b-value DTI techniques into clinical practice.

**Graphical Abstract:**

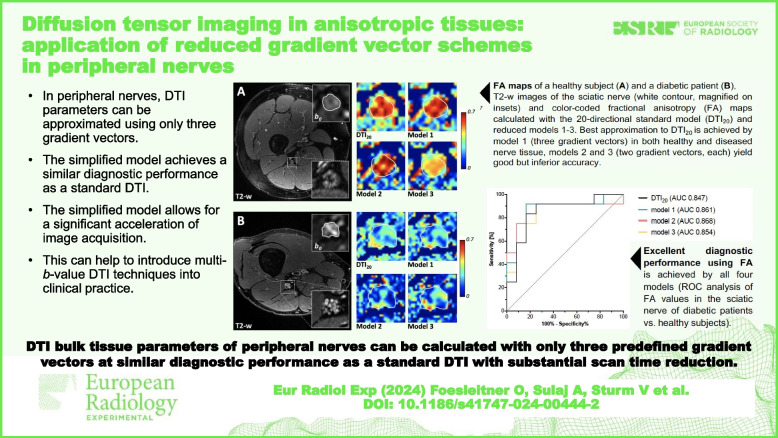

**Supplementary Information:**

The online version contains supplementary material available at 10.1186/s41747-024-00444-2.

## Background

Diffusion tensor imaging (DTI) and tractography are established noninvasive imaging techniques to assess neuronal fiber integrity and directionality in the central nervous system. Based on Brownian molecular motion, pathology-specific changes in diffusivity can be detected and derived from DTI scalars and thus be used as biomarkers [1]. The precision and clinical value of DTI metrics are determined by several technical parameters, particularly by a selection of *b*-values and the gradient vector scheme. Recent DTI sequences using multiple *b*-values and high numbers of diffusion directions seek to exploit non-Gaussian diffusion effects to reveal novel microstructural tissue characteristics [2–4]. However, these sequences require substantially longer acquisition times and are therefore currently not feasible for clinical routine.

Besides the central nervous system, other organ systems like the peripheral nervous system have emerged as highly promising areas for the application of DTI [5–7]. Despite fundamental differences in tissue architecture, DTI sequence parameters are commonly adopted from the central nervous system. In contrast to the complex fiber architecture of the brain, peripheral nerves typically follow a monodirectional course which can be aligned to the *z*-axis of the magnetic resonance imaging (MRI) scanner by correct positioning. Hence, it seems questionable if multidirectional gradient vector schemes in peripheral nerve DTI are necessary when probing bulk tissue characteristics such as fractional anisotropy (FA). As scan time increases linearly with the number of either diffusion directions or *b*-values, reduced gradient vector schemes appear highly desirable to implement advanced diffusion tissue characteristics such as non-Gaussian diffusion in clinical practice [8]. Taking the example of diabetic neuropathy as a common peripheral nerve disorder, novel quantitative biomarkers derived from non-Gaussian diffusion could provide complementary tissue information and thereby improve diagnostic specificity [8].

Taking the simplified geometrical constraints of peripheral nerves into consideration, this study aims to investigate the accuracy of measurements obtained using DTI with diffusion directions reduced to the nerve’s main axes compared to a standard multidirectional DTI sequence as reference, and possible influencing factors that may limit their applicability.

## Methods

### Study design and data acquisition

This proof-of-concept study was conducted in accordance with the Declaration of Helsinki and approved by the local institutional Ethics Committee (S-398/2012, S-682/2016, S-499/2019). Written informed consent was obtained from all participants. Subject details can be retrieved from Table 1. The diabetic patient group was further characterized by the following clinical parameters: disease duration 13.9 ± 11.3 years, body mass index 28.8 ± 3.5 kg/m^2^, glycated hemoglobin–HbA1c 7.5 ± 1.1%, neuropathy disability score 3.3 ± 2.2, neuropathy symptom score 6.1 ± 2.9 (mean ± standard deviation). Only patients with no other disease possibly causing peripheral neuropathy were eligible.

**Table 1:** Demographics of study participants

	Healthy subjects	Diabetic patients	Age-matched controls
Number of participants	10	12	12
Age, years, median (range)	34.0 (25–41)	68.5 (54–73)	67.5 (59–73)
Sex (male/female)	5/5	10/2	10/2
Nerve regions acquired	Tibial nerve (distal thigh)	Tibial nerve (distal thigh)	Tibial nerve (distal thigh)
	Tibial nerve (proximal calf)		
	Median nerve (upper arm)		
	Radial nerve (upper arm)		
	Ulnar nerve (upper arm)		

MRI examinations were performed on a 3-T scanner (Magnetom TrioTim or Prismafit, Siemens Healthineers, Erlangen, Germany) between 01/2016 and 12/2019 at a single academic institution. Single-shot echo-planar imaging DTI sequences were acquired at the distal thigh in all 34 participants as well as at the proximal calf and the mid-upper arm in 10 younger healthy volunteers. The lower extremity was examined in a supine position with a 15-channel transmit-receive knee coil (Siemens Healthineers). The upper arm was examined in a prone position with a 16-channel receive-only multipurpose flex coil (Variety, Noras MRI Products, Hoechberg, Germany). For anatomical delineation, an axial fat-saturated T2-weighted sequence was acquired at each level. Details of sequence parameters are listed in Table 2.

**Table 2:** Parameters of diffusion tensor imaging and T2-weighted sequences

	Diffusion tensor imaging		T2-weighted sequences	
Scanner	Prisma^fit^	TimTrio	Prisma^fit^	TimTrio
Repetition time (ms)	4,000	4,000	6,969	8,150
Echo time (ms)	87	92.8	54	54
Field of view	160 × 160 mm^2^	160 × 160 mm^2^	140 × 140 mm^2^	160 × 160 mm^2^
Matrix	128 × 128	128 × 128	512 × 358	512 × 333
Slice thickness (mm)	4.0	4.0	3.5	3.5
Number of slices	18	18	35	41
Slice gap (mm)	0.4	1.2	0.35	0.35
Number of averages	3	3	3	2
Parallel imaging	GRAPPA	GRAPPA	GRAPPA	GRAPPA
Acceleration factor	2	3	2	2
Acquisition time (min:s)	4:34	4:32	04:22	5:29
Fat saturation	SPAIR	SPAIR	Spectral	Spectral
*b* value	0, 1,000 s/mm^2^	0, 1,000 s/mm^2^	−	−
Diffusion gradients	20	20	−	−

*GRAPPA* Generalized autocalibrating partial parallel acquisition, *SPAIR* Spectral adiabatic inversion recovery

To probe the applicability of reduced gradient vector schemes for bulk tissue DTI metrics, diffusion parameter values (see below) determined by the conventional DTI sequence comprising 20 diffusion directions (DTI_20_) were defined as the “reference standard.” Parameter values determined by the simplified, reduced gradient vector schemes were then compared to the gold standard. This procedure was performed under physiological conditions in a cohort of young and older healthy individuals. Furthermore, to test the simplified approach in a broader and more general context under exemplary pathological conditions, we used the simplified approach in a cohort of patients with diabetic neuropathy who exhibited well-known phenotypical fascicular nerve lesions on T2-weighted imaging (Table 1).

### Data processing and analysis

The target nerves were first identified on T2-weighted images and regions of interest along the nerves were manually drawn on corresponding b_0_ images on all 18 slices per imaging region (ImageJ, version 2.3.0/1.53f). Spatial misalignment of nerves on b_1000_ images was corrected manually.

Apparent diffusion coefficient (ADC) and FA maps of the original DTI_20_ dataset were generated on-site by the integrated postprocessing software (Siemens, Healthineers), while axial and radial diffusivity (AD, RD) maps were calculated using a custom-written Matlab routine (R2015a, MathWorks, Natick, MA, USA). The cross-sectional area and mean signal intensity of each region of interest were extracted.

Based on DTI_20_, three reduced gradient vector datasets were built in which the vector closest to the scanner’s *z*-axis was fixed as the main eigenvector representing the axial diffusion component. In model 1, the radial diffusion components were predefined as the vectors in line with the *x*- and *y*-axes. In models 2 and 3, only one of these two predefined radial eigenvectors was included (see also Supplementary Material). DTI parameters derived from the simplified models are henceforth referred to as AD_pseudo_, RD_pseudo_, ADC_pseudo_, and FA_pseudo_.

Accuracy was measured as the absolute difference between DTI parameters calculated with models 1, 2, or 3 and those derived from DTI_20_. Precision, on the other hand, was assessed using the Brown-Forsythe test by determining the median value of each parameter of the 18 slices per imaging region for each subject and calculating the within-subject variance for each model, in line with previous recommendations [9]. Differences among the models were assessed using the Friedman test with Dunn’s correction for multiple comparisons, while the Kruskal–Wallis test was applied to investigate differences among study groups. Additionally, the Bland–Altman analysis was used to evaluate the accuracy of models 1, 2, and 3. Spearman’s correlation coefficients were used to assess the influence of nerve angulation and intraneural connective tissue. Angulation of the nerve with respect to the *z*-axis was calculated using the connecting vector between the region of interest center of two neighboring slices. Intraneural connective tissue was quantified on T2-weighted images using an individual threshold based on the corresponding signal intensity of surrounding fat tissue.

The area under the receiver operating characteristic curve was used to assess diagnostic performance between patients and matched controls. Statistical tests were conducted using SPSS version 27 (IBM, Armonk, NY, USA) and Prism version 8 (GraphPad Software, Boston, MA, USA). The threshold of significance was set at *p* < 0.05.

## Results

### Accuracy of measurements

To assess the general feasibility of simplified gradient vector schemes, we first calculated FA_pseudo_, ADC_pseudo_, RD_pseudo_, and AD_pseudo_ and compared the results to those obtained from DTI_20_.

**Fig. 1:**
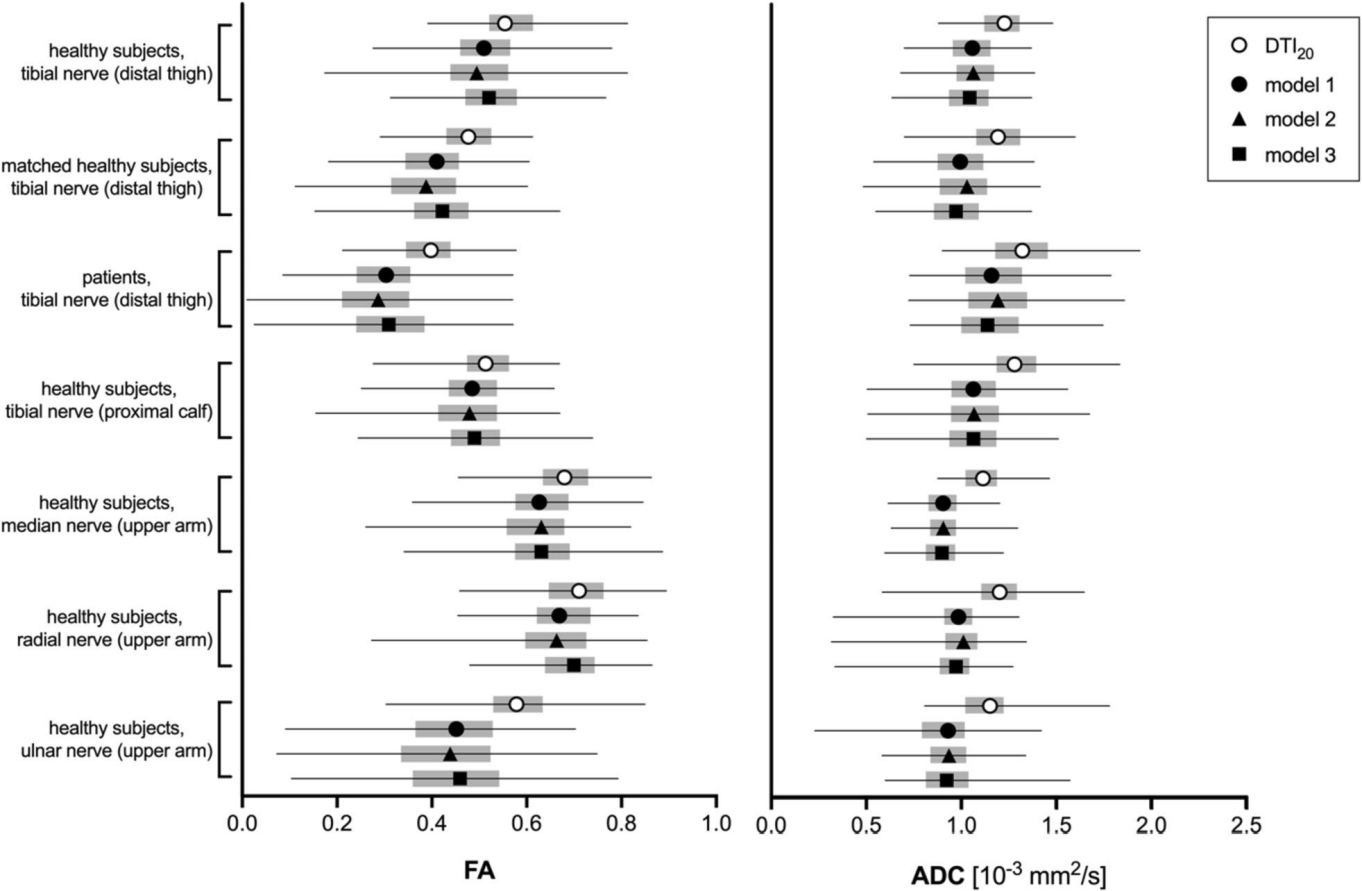
Accuracy of different fractional anisotropy (FA) and apparent diffusion coefficient (ADC) calculation models. Forest plots of absolute FA values (left side) and ADC (right side) show the accuracy of models 1, 2, and 3 compared to the 20-directional standard model (DTI_20_). Symbols indicate the median, horizontal lines depict the range, and gray boxes show the interquartile range

Absolute FA_pseudo_ values derived from models 1, 2, and 3 were lower than those from DTI_20_ in all nerves and groups (*p* < 0.001) except for the radial nerve in model 3 (*p* = 0.393). Median absolute differences were ranging from -0.029 to -0.117 for model 1, from - 0.034 to -0.119 for model 2, and from -0.015 to -0.114 for model 3 (Fig. 1; Supplementary Table S1). Similarly, absolute ADC_pseudo_ values were consistently lower in models 1–3 compared to DTI_20_ (*p* < 0.001) with differences ranging from -0.125 mm^2^/s to -0.246 mm^2^/s (Fig. 1). Bland–Altman plots showed a tendency towards better accuracy with higher absolute FA and ADC values for models 2 and 3 but not so for model 1 (Supplementary Figs. S1 and S2). Models 1, 2, and 3 yielded lower AD_pseudo_ values than DTI_20_ in all comparisons with median differences of -0.492 to -0.961 × 10^-3^ mm^2^/s (*p* < 0.001 each). RD_pseudo_ tended to be lower in models 1, 2, and 3 with median differences between -0.071 and 0.030 × 10^-3^ mm^2^/s (Supplementary Fig. S3).

### Precision of measurements

As the reliability and diagnostic value of a measurement highly depend on its consistency, we further assessed the individual precision of the simplified diffusion datasets compared to DTI_20_.

**Fig. 2:**
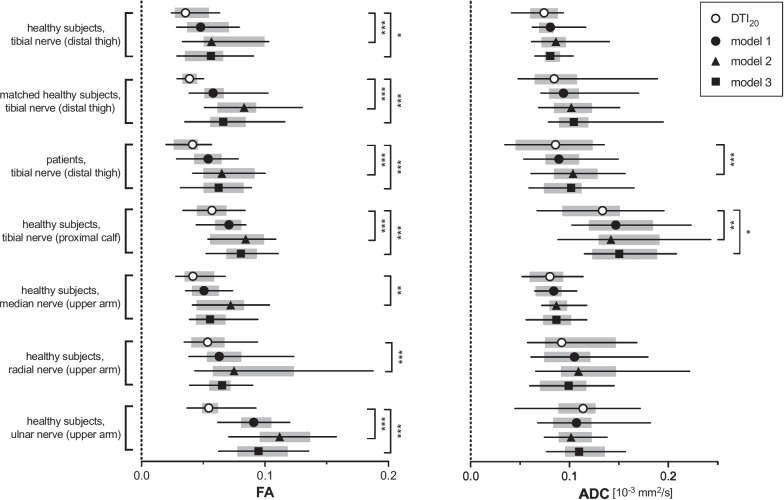
Precision of different fractional anisotropy (FA) and apparent diffusion coefficient (ADC) calculation models. Forest plots of within-subject variance of fractional anisotropy (FA, left side) and apparent diffusion coefficient (ADC, right side) show the precision of models 1, 2, and 3 compared to the 20-directional standard model (DTI_20_). Symbols indicate the median, horizontal lines depict the range, and gray boxes show the interquartile range. Significant differences between models 1, 2, and 3 to DTI_20_ are indicated with asterisks (*p < 0.05; **p < 0.01; **p < 0.001)

The within-subject variance of FA_pseudo_ values tended to be lower in DTI_20_ than in FA_pseudo_ from models 1, 2, and 3 (Supplementary Table S2). Differences in variance were small and only significant in model 2 (7/7 cases) and model 3 (5 of 7 cases; Fig. 2). Individual variance of ADC_pseudo_ values was similar to DTI_20_ with model 1 in 7 of 7 cases, with model 2 in 5 of 7 cases, and with model 3 in 6 of 7 cases. AD_pseudo_ values showed consistently lower within-subject variance in models 1, 2, and 3 compared to DTI_20_ (*p* < 0.001 each; Supplementary Fig. S4). Individual variance of RD_pseudo_ compared to DTI_20_ was equal or better with model 1 in 7 of 7 cases, with model 2 in 5 of 7 cases, and with model 3 in 5 of 7 cases than with DTI_20_.

### Diagnostic performance

While simplified datasets, especially model 1, seem to provide similar reliability measures as DTI_20_, their accuracy appears lower. This raises the important question of whether this precludes accurate discrimination between healthy and diseased nerves.

**Fig. 3:**
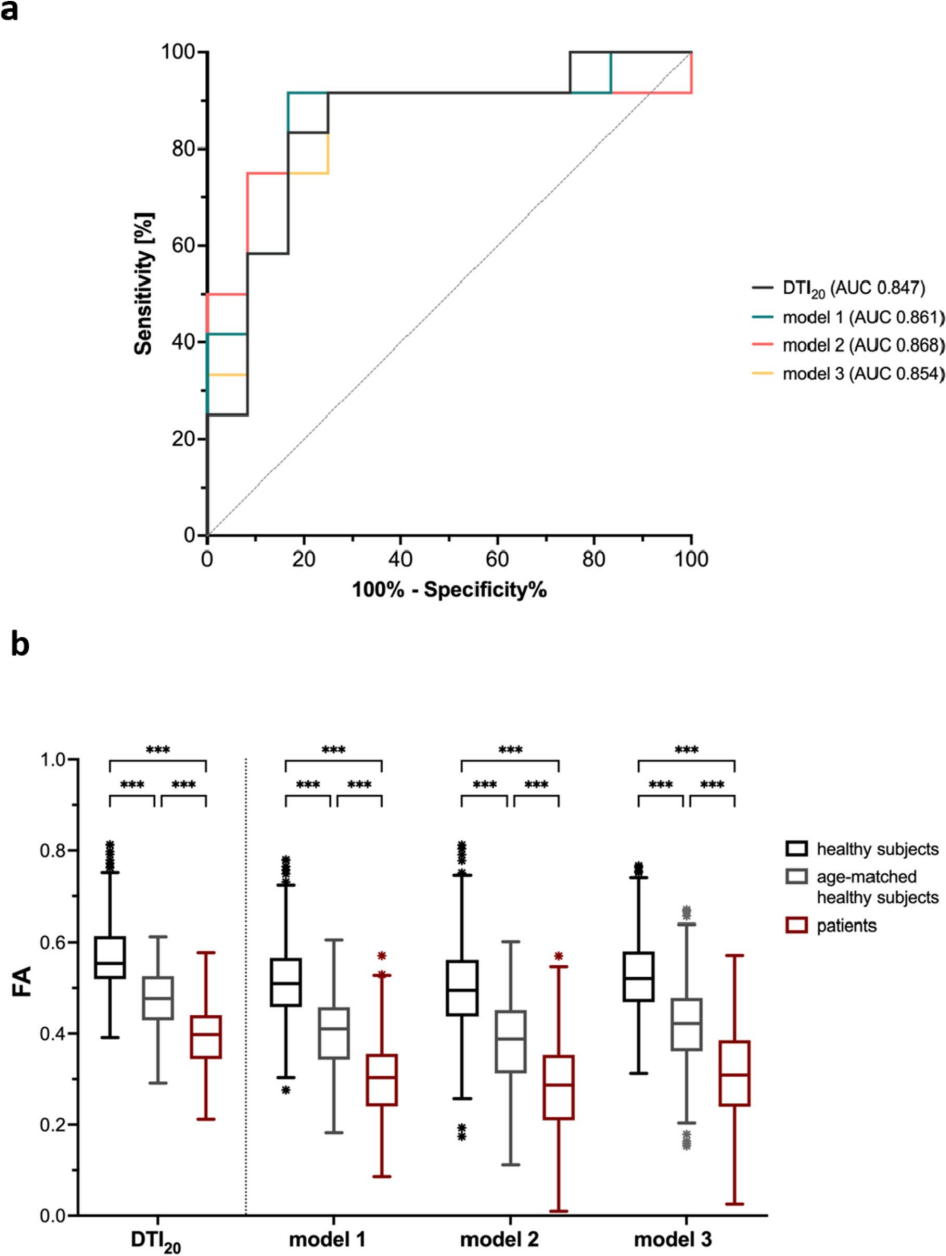
Diagnostic performance based on different fractional anisotropy (FA) calculation models. Receiver operating characteristic analysis (**a**) and box-and-whisker plots (**b**) of FA measured in the tibial nerve at the distal thigh show excellent discrimination between diabetic patients and healthy subjects by all four analyzed models. Data are medians (lines in boxes), 25th to 75th percentiles (bottom and top of boxes), and ranges (Tukey whiskers)

**Fig. 4:**
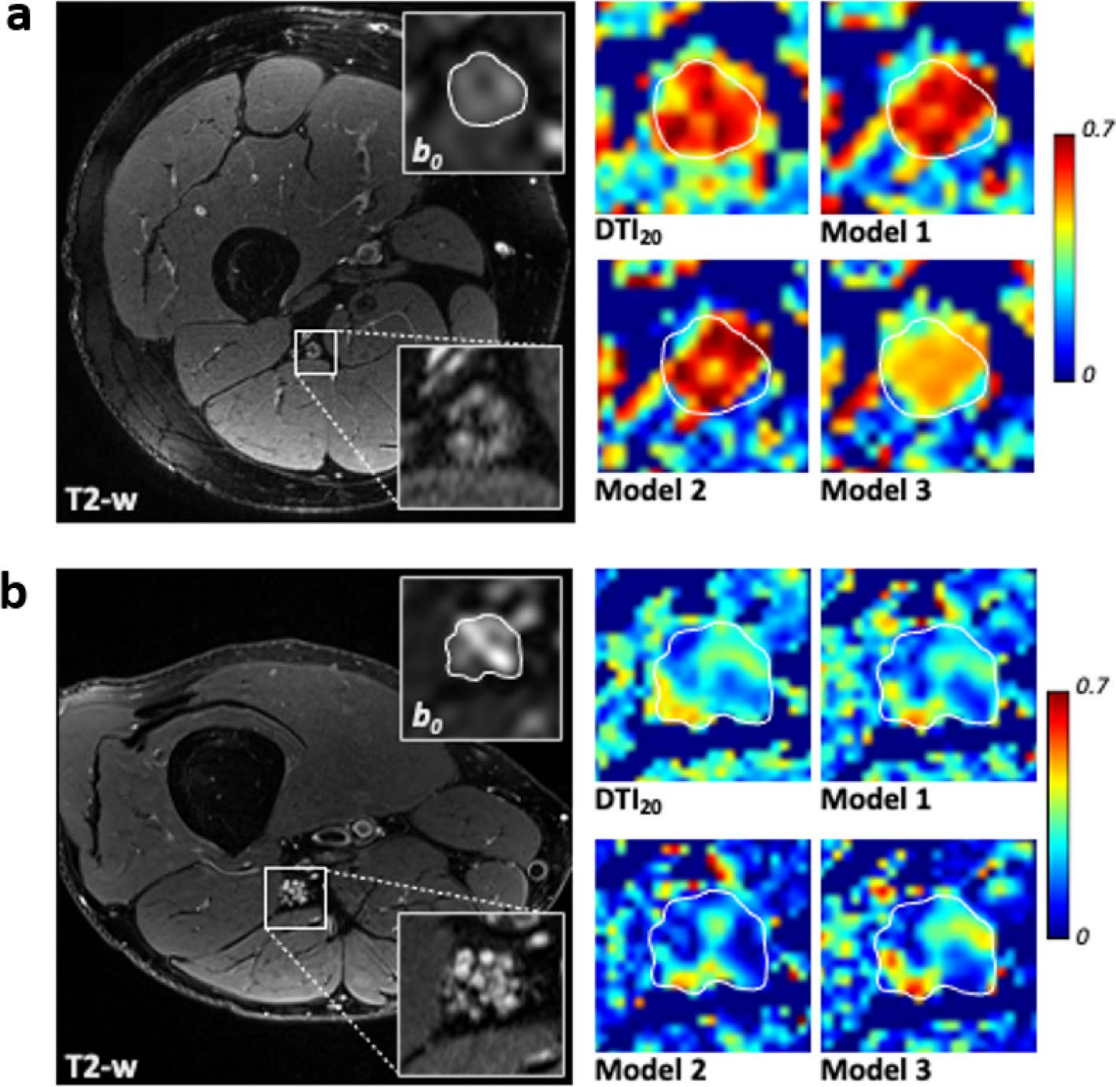
Fractional anisotropy (FA) map reconstructions in healthy and diabetic patients. Representative images of a healthy subject (**a**) and a diabetic patient (**b**) showing T2-weighted images of the distal sciatic nerve and corresponding color-coded fractional anisotropy (FA) maps calculated with the 20-directional standard model (DTI_20_) and the reduced models 1, 2, and 3. Segmentation of the nerve portion is indicated by a white contour. Insets show a magnification of the nerve. Model 1 achieves the best approximation to DTI_20_ in both healthy and diseased nerve tissue, while models 2 and 3 yield good but inferior accuracy

FA values from DTI_20_ as well as FA_pseudo_ values from models 1, 2, and 3 were clearly lower in diabetic patients than in young or age-matched controls (*p* < 0.001, Fig. 3b). ROC analysis revealed excellent discrimination between patients and age-matched controls by all models achieving comparable values of areas under the receiver operating characteristics curve of 0.847 (DTI_20_, *p* = 0.004), 0.854 (model 3, *p* = 0.003), 0.861 (model 1, *p* = 0.003), and 0.868 (model 2, *p* = 0.002; Fig. 3a). Furthermore, patients had increased ADC or ADC_pseudo_ and RD or RD_pseudo_ values compared to either control group (*p* < 0.001) in all models, while AD_pseudo_ from models 1, 2, and 3 was only slightly higher in patients (*p* < 0.001 and *p* = 0.003; Supplementary Fig. S5). Figure 4 shows FA maps derived from DTI_20_ and models 1, 2, and 3 in a representative diabetic patient and healthy participant.

### Influence of intraneural connective tissue and nerve angulation

The proportion of intraneural connective tissue was negatively correlated with absolute FA values in DTI_20_ and FA_pseudo_ values in all simplified datasets, showing the highest dependence in young healthy subjects (*r* = -0.49 to -0.32, *p* < 0.001 each; Supplementary Tables S3 and S4; Supplementary Fig. 6a). Accuracy was not significantly correlated with connective tissue proportions. Nerve angulation with respect to the scanner’s *z*-axis ranged from a median of 5.79° (range 0.31–15.8) in the median nerve to 13.30° (range 2.22–30.50) in the radial nerve (Supplementary Table S3). Only weak associations were found between nerve angulation and absolute FA or FA_pseudo_ values or accuracy (Supplementary Table 5 and Fig. S6b).

## Discussion

In this proof-of-concept study, we investigated whether simplified DTI models could yield similar quantitative diffusion tissue parameters of peripheral nerves as a standard DTI sequence with 20 gradient vectors. Our results show that, indeed, models using only three or even two predefined diffusion directions can be used to calculate diffusion biomarkers including FA, ADC, AD, and RD at high diagnostic accuracy. Neither nerve angulation nor the amount of intraneural connective tissue had a relevant negative effect on the accuracy or precision of these simplified models. Diagnostic accuracy to detect diabetic neuropathy was equally excellent with the simplified models as with the multidirectional standard model. It is important to note, however, that in this study, diabetic neuropathy only served as a prime example of peripheral nerve pathology in general to test the simplified technical concept, being itself already extensively studied by MRI [10–14].

Although often providing valuable information [5, 11, 15–17], DTI sequences are only rarely part of routine clinical MR-neurography protocols. An important reason may be a lack of consensus on optimal sequence parameters [18], or extended acquisition times especially if complex tissue characteristics are of interest including the use of multiple *b*-values [19–21].

The number of diffusion gradient vectors is one main factor determining acquisition time. However, complex vector schemes with a high number of gradient directions are only needed when probing very complex tissues like crossing or bending fibers in the brain. Peripheral nerves, on the contrary, mainly follow a monodirectional course and are thus approximately axially symmetric. Although it is known that at least 6 gradient sets are required for a true quantitative measurement of ADC due to the rotational variance of perpendicular gradient sets [22], we hypothesized that a simplified set of gradients using the principal diffusion direction (*i.e.,* the nerve’s longitudinal axis) as being in line with the *z*-axis of the MR scanner could be sufficient for clinical diagnostic purposes. This would allow to radically decrease the required number of vectors for quantitative DTI metrics to three or even two, assuming that radial diffusivity could be expressed by either eigenvector ε_2_ or ε_3_. A similar concept using a priori knowledge of the primary diffusion direction to simplify data acquisition was shown to be feasible for diffusion kurtosis imaging in an experimental setting [23].

In practice, our study shows that two-vector models yield only inconsistently good estimates of diffusion metrics and may therefore constitute an oversimplification, particularly in lower absolute FA and ADC values. The three-vector model including both perpendicular radial diffusion directions, on the other hand, achieved significantly better accuracy than the 2-vector models and even equal precision as the 20-vector model. Absolute values of DTI metrics calculated with the reduced datasets were mostly lower than with the full 20-vector model, most likely due to a suboptimal selection of the main eigenvector and thus a decrease in AD estimates. Given the linear relation between the number of diffusion directions and scan time—when not additionally compensating for signal-to-noise ratio loss—the reduction from 20 to 3 vectors leads to a scan time reduction of 84% (*i.e.,* an acquisition time of less than one minute instead of 4:30 min:s in our case). This opens the possibility to add other diffusion aspects such as multiple b-value techniques for a more in-depth understanding of tissue microarchitecture [20, 21, 23] or simply to substantially accelerate the examination time.

As shown in the example of diabetic neuropathy, advanced diffusion metrics could improve diagnostic performance by introducing novel quantitative parameters [8]. This could be of particular value in diffuse peripheral neuropathies with ambiguous clinical presentation, which often show similar patterns on conventional T2-weighted sequences.

Regarding factors potentially influencing read-out parameters in a negative way, we found that smaller deviations of nerve angulation seemed to have a negligible effect on the accuracy of the simplified diffusion models. Similarly, the accuracy of the models was not found to be strongly affected by the amount of intraneural connective tissue. Instead, we found that absolute FA values decreased with more intraneural connective tissue in all models including the standard 20-vector model. Given the currently limited spatial resolution of diffusion sequences, this raises the question of whether partial volume effects may substantially contribute to the reduction of FA found in many peripheral neuropathies.

This study comes with some limitations. First, our study population was limited in size albeit carefully selected to be homogenous. Secondly, we focused on diabetic neuropathy as a well-known structural nerve disease to validate our simplified models in a pathological setting. Although this yielded excellent results, it will be necessary to investigate whether our concept also applies to other peripheral nerve disorders to pave the way for broader clinical applicability. Thirdly, the main nerve axes were approximated to be in line with the MRI scanner’s axes, which proved to generate accurate results within the range of angulation measured. Yet, higher deviations from this assumption would expectedly lead to more inaccurate results, *e.g.,* if patients do not tolerate adequate positioning. Finally, this study investigated whether simplified models containing only two or three gradient vectors were able to generate and approximate gross diffusion parameters such as FA. For a detailed assessment of nerve continuity and visualization with tractography, on the other hand, more complex models would be favorable.

In conclusion, this study shows that in highly anisotropic tissues such as peripheral nerves, bulk tissue parameters can be accurately estimated with a simplified diffusion model using only three predefined gradient vectors along the scanner’s main axes—without apparent diagnostic drawbacks. The resulting sixfold scan time reduction compared to a standard DTI sequence with 20 directions could instead be invested in accelerated examinations or in multiple *b*-value acquisitions allowing for novel MR-biomarkers based on non-Gaussian diffusion.

### Supplementary Information

**Additional file 1: Suppl. Table 1.** Values of fractional anisotropy (FA), apparent diffusion coefficient (ADC), axial diffusivity (AD), and radial diffusivity (RD). Values are median (IQR). ADC [mm^2^/s], AD [10–3 mm^2^/s], RD [10–3 mm^2^/s]. **Suppl. Table 2.** Standard deviation (SD) of fractional anisotropy (FA), apparent diffusion coefficient (ADC), axial diffusivity (AD), and radial diffusivity (RD) values. Values are median (min-max). **Suppl. Table 3.** Values of nerve angulation and intraneurial connective tissue across regions. Values are median (min-max). **Suppl. Table 4a**. Correlation analysis of absolute fractional anisotropy values and intraneurial connective tissue. *p* values < .05 are marked in bold letters. r, Spearman's correlation coefficient. **Suppl. Table 5a.** Correlation analysis of absolute fractional anisotropy values and nerve angulation. *p* values < .05 are marked in bold letters. r, Spearman's correlation coefficient. **Suppl. Table 4b**. Correlation analysis of accuracy of fractional anisotropy values and intraneurial connective tissue. *p* values < .05 are marked in bold letters. r, Spearman's correlation coefficient. **Suppl. Table 5b**. Correlation analysis of accuracy of fractional anisotropy values and nerve angulation. *p* values <. 05 are marked in bold letters. r, Spearman's correlation coefficient. **Supplementary Figure 1.** Accuracy of FA as assessed by Bland-Altman plots. Bland-Altman plots showing the limits of difference (± 2 SD) in healthy subjects (upper panel), diabetic patients (middle panel) and age-matched controls (lower panel) between paired values of fractional anisotropy (FA) calculated with 20-vector DTI and model 1 (A, D, G), model 2 (B, E, H), or model 3 (C, F, I) in the tibial nerve at thigh level. **Supplementary Figure 2.** Accuracy of ADC as assessed by Bland-Altman plots. Bland-Altman plots showing the limits of difference (± 2 SD) in healthy subjects (upper panel), diabetic patients (middle panel) and age-matched controls (lower panel) between paired values of apparent diffusion coefficient (ADC) calculated with 20-vector DTI and model 1 (A, D, G), model 2 (B, E, H), or model 3 (C, F, I) in the tibial nerve at thigh level. **Supplementary Figure 3.** Accuracy of different AD and RD calculation models. Forest plots of absolute axial diffusivity (AD, left side) and radial diffusivity (right side) show the accuracy of models 1-3 compared to the 20-directional standard model (DTI20). Symbols indicate the median, horizontal lines depict the range, and gray boxes show the interquartile range. **Supplementary Figure 4.** Precision of different AD and RD calculation models. Forest plots of within-subject precision of axial diffusivity (AD, left side) and radial diffusivity (RD, right side) show the precision of models 1-3 compared to the 20-directional standard model (DTI20). Standard deviation is plotted on the x-axes. Symbols indicate the median, horizontal lines depict the range, and gray boxes show the interquartile range. Significant differences between models 1-3 to DTI20 are indicated with asterisks (* *p *< .05; ** *p *< .01, ** *p *< .001). **Supplementary Figure 5.** Group differences based on different ADC, AD and RD calculation models. Box-and-whisker plots of apparent diffusion coefficient (ADC) and axial and radial diffusivity (AD, RD) measured in the tibial nerve at the distal thigh show group differences between diabetic patients and healthy subjects using the four analyzed models. Data are medians (lines in boxes), 25th to 75th percentiles (bottom and top of boxes), and ranges (Tukey whiskers). **Supplementary Figure 6.** FA dependence on intraneurial connective tissue and nerve angulation. Scatter plot of fractional anisotropy (FA) measured in the tibial nerve at the distal thigh in young healthy subjects in relation to percentage of intraneurial connective tissue shows a negative correlation in all four examined models (A), while no clear dependence was detected between FA accuracy and nerve angulation (B).

## Data Availability

The datasets used and/or analyzed during the current study are available from the corresponding author on reasonable request.

## Abbreviations

AD: Axial diffusivity
ADC: Apparent diffusion coefficient
ADCpseudo: Apparent diffusion coefficient calculated with reduced models
ADpseudo: Axial diffusivity calculated with reduced models
DTI: Diffusion tensor imaging
FA: Fractional anisotropy
FApseudo: Fractional anisotropy calculated with reduced models
RD: Radial diffusivity
RDpseudo: Radial diffusivity calculated with reduced models
