# Supplementary material for: Diffusion tensor imaging in anisotropic tissues: application of reduced gradient vector schemes in peripheral nerves
Source: Eur Radiol Exp. 2024 Apr 2;8:37. doi: 10.1186/s41747-024-00444-2 (PMC10984907; doi:10.1186/s41747-024-00444-2)
Supplement: Supplementary file 1 — Additional file 1: Suppl. Table 1. Values of fractional anisotropy (FA), apparent diffusion coefficient (ADC), axial diffusivity (AD), and radial diffusivity (RD). Values are median (IQR). ADC [mm2/s], AD [10–3 mm2/s], RD [10–3 mm2/s]. Suppl. Table 2. Standard deviation (SD) of fractional anisotropy (FA), apparent diffusion coefficient (ADC), axial diffusivity (AD), and radial diffusivity (RD) values. Values are median (min-max). Suppl. Table 3. Values of nerve angulation and intraneurial connective tissue across regions. Values are median (min-max). Suppl. Table 4a. Correlation analysis of absolute fractional anisotropy values and intraneurial connective tissue. p values < .05 are marked in bold letters. r, Spearman's correlation coefficient. Suppl. Table 5a. Correlation analysis of absolute fractional anisotropy values and nerve angulation. p values < .05 are marked in bold letters. r, Spearman's correlation coefficient. Suppl. Table 4b. Correlation analysis of accuracy of fractional anisotropy values and intraneurial connective tissue. p values < .05 are marked in bold letters. r, Spearman's correlation coefficient. Suppl. Table 5b. Correlation analysis of accuracy of fractional anisotropy values and nerve angulation. p values <. 05 are marked in bold letters. r, Spearman's correlation coefficient. Supplementary Figure 1. Accuracy of FA as assessed by Bland-Altman plots. Bland-Altman plots showing the limits of difference (± 2 SD) in healthy subjects (upper panel), diabetic patients (middle panel) and age-matched controls (lower panel) between paired values of fractional anisotropy (FA) calculated with 20-vector DTI and model 1 (A, D, G), model 2 (B, E, H), or model 3 (C, F, I) in the tibial nerve at thigh level. Supplementary Figure 2. Accuracy of ADC as assessed by Bland-Altman plots. Bland-Altman plots showing the limits of difference (± 2 SD) in healthy subjects (upper panel), diabetic patients (middle panel) and age-matched controls (lower panel) between pai [file 41747_2024_444_MOESM1_ESM.docx]

**Diffusion tensor imaging in anisotropic tissues: application of reduced gradient vector schemes in peripheral nerves**

**ELECTRONIC SUPPLEMENTARY MATERIAL**

**Formulae and calculation of DTI parameters**

The fractional anisotropy (FA) was calculated according to:

$$FA= \sqrt{\frac{{(\lambda_{1}-\lambda_{2})}^{2}+{(\lambda_{1}-\lambda_{3})}^{2}+{(\lambda_{2}-\lambda_{3})}^{2}}{2 ({\lambda_{1}}^{2}+{\lambda_{2}}^{2}+{\lambda_{3}}^{2})}}$$

where λ_1-3_ represent the eigenvalues in descending order of their magnitude in the full 20-gradient vector model (DTI_20_) with λ_1_ > λ_2_ > λ_3_, which means the eigenvectors ε_1-3_ are retrospectively selected among the 20 vectors. For models 1-3, eigenvalues were derived from predefined eigenvectors ε_1-3_. In all three models, ε_1_ was set at the vector closest to the z-axis as the presumed longitudinal axis of the peripheral nerve and thus representing axial diffusivity (AD, 10^-3^ mm^2^/s). Due to slightly different sequence parameters in the two MRI scanners, ${}_{1}=\left[ \begin{matrix} -0.12 \\ -0.23 \\ -0.96 \end{matrix} \right]$ for healthy subjects and ${}_{1}=\left[ \begin{matrix} -0.09 \\ 0.26 \\ -0.96 \end{matrix} \right]$ for patients. In model 1, the radial eigenvectors were fixed at ${}_{2}=\left[ \begin{matrix} 1 \\ 0 \\ 0 \end{matrix} \right]$ and ${}_{3}=\left[ \begin{matrix} 0 \\ 1 \\ 0 \end{matrix} \right]$ and thus in line with the x- and y-axis, respectively. The radial eigenvectors were set at ${}_{2}={}_{3}=\left[ \begin{matrix} 1 \\ 0 \\ 0 \end{matrix} \right]$ in model 2 and at ${}_{2}={}_{3}=\left[ \begin{matrix} 0 \\ 1 \\ 0 \end{matrix} \right]$ in model 3, following the presumption that radial diffusivity (RD) in peripheral nerves is approximately uniform. RD (10^-3^ mm^2^/s) was calculated as$\frac{(\lambda_{2}+\lambda_{3})}{2}$ . The apparent diffusion coefficient (ADC, 10^-3^ mm^2^/s) was calculated according to:

$$ADC=-\frac{1}{b}*\ln\left( \frac{S_{x}}{S_{0}} \right)$$

where S_0_ is the signal magnitude at *b* = 0 and S_x_ at *b* = 1000 s/mm^2^.

**Supplementary Tables:**

**Supplementary Table 1**

|  |  | | | |  | | |  | | |  | | |  | | |  | | |
| --- | --- | --- | --- | --- | --- | --- | --- | --- | --- | --- | --- | --- | --- | --- | --- | --- | --- | --- | --- |
|  |  | | **DTI_20_** | | | **Model 1** | | | | **Model 2** | | | **Model 3** | | |  | | |  |
|  | |  | |  | | |  | |  | | |  | | |  | | |  |  |
| **healthy subjects** | | tibial nerve (distal thigh) | | | | |  | |  | | |  | | |  | | |  |  |
|  | | FA | | 0.55 (0.52, 0.61) | | | 0.51 (0.46, 0.57) | | 0.50 (0.44, 0.56) | | | 0.52 (0.47, 0.58) | | |  | | |  |  |
|  | |  | |  | | |  | |  | | |  | | |  | | |  |  |
|  | | ADC | | 1.23 (1.12, 1.31) | | | 1.06 (0.95, 1.16) | | 1.06 (0.97, 1.18) | | | 1.04 (0.93, 1.15) | | |  | | |  |  |
|  | |  | |  | | |  | |  | | |  | | |  | | |  |  |
|  | | AD | | 2.24 (2.10, 2.39) | | | 1.74 (1.58, 1.91) | | 1.74 (1.58, 1.91) | | | 1.74 (1.58, 1.91) | | |  | | |  |  |
|  | |  | |  | | |  | |  | | |  | | |  | | |  |  |
|  | | RD | | 0.77 (0.67, 0.87) | | | 0.73 (0.61, 0.81) | | 0.73 (0.64, 0.81) | | | 0.71 (0.60, 0.79) | | |  | | |  |  |
|  | |  | |  | | |  | |  | | |  | | |  | | |  |  |
| **age-matched controls** | | tibial nerve (distal thigh) | | | | |  | |  | | |  | | |  | | |  |  |
|  | | FA | | 0.48 (0.43, 0.53) | | | 0.41 (0.34, 0.46) | | 0.39 (0.31, 0.45) | | | 0.42 (0.36, 0.48) | | |  | | |  |  |
|  | |  | |  | | |  | |  | | |  | | |  | | |  |  |
|  | | ADC | | 1.19 (1.08, 1.31) | | | 0.99 (0.87, 1.12) | | 1.03 (0.88, 1.14) | | | 0.97 (0.85, 1.10) | | |  | | |  |  |
|  | |  | |  | | |  | |  | | |  | | |  | | |  |  |
|  | | AD | | 2.24 (2.09, 2.39) | | | 1.49 (1.32, 1.64) | | 1.49 (1.32, 1.64) | | | 1.49 (1.32, 1.64) | | |  | | |  |  |
|  | |  | |  | | |  | |  | | |  | | |  | | |  |  |
|  | | RD | | 0.79 (0.69, 0.92) | | | 0.75 (0.64, 0.87) | | 0.78 (0.66, 0.92) | | | 0.72 (0.61, 0.83) | | |  | | |  |  |
|  | |  | |  | | |  | |  | | |  | | |  | | |  |  |
| **patients** | | tibial nerve (distal thigh) | | | | |  | |  | | |  | | |  | | |  |  |
|  | | FA | | 0.40 (0.34, 0.44) | | | 0.30 (0.24, 0.36) | | 0.29 (0.21, 0.35) | | | 0.31 (0.24, 0.39) | | |  | | |  |  |
|  | |  | |  | | |  | |  | | |  | | |  | | |  |  |
|  | | ADC | | 1.32 (1.18, 1.46) | | | 1.16 (1.02, 1.32) | | 1.19 (1.03, 1.35) | | | 1.14 (1.00, 1.31) | | |  | | |  |  |
|  | |  | |  | | |  | |  | | |  | | |  | | |  |  |
|  | | AD | | 2.17 (2.03, 2.48) | | | 1.60 (1.43, 1.79) | | 1.60 (1.43, 1.79) | | | 1.60 (1.43, 1.79) | | |  | | |  |  |
|  | |  | |  | | |  | |  | | |  | | |  | | |  |  |
|  | | RD | | 0.93 (0.82, 1.09) | | | 0.93 (0.82, 1.12) | | 0.97 (0.84, 1.15) | | | 0.93 (0.78, 1.09) | | |  | | |  |  |
|  | |  | |  | | |  | |  | | |  | | |  | | |  |  |
| **healthy subjects** | | tibial nerve (prox. calf) | | | | |  | |  | | |  | | |  | | |  |  |
|  | | FA | | 0.51 (0.47, 0.56) | | | 0.49 (0.43, 0.54) | | 0.48 (0.41, 0.54) | | | 0.49 (0.44, 0.55) | | |  | | |  |  |
|  | |  | |  | | |  | |  | | |  | | |  | | |  |  |
|  | | ADC | | 1.28 (1.18, 1.40) | | | 1.06 (0.95, 1.19) | | 1.07 (0.94, 1.20) | | | 1.06 (0.94, 1.19) | | |  | | |  |  |
|  | |  | |  | | |  | |  | | |  | | |  | | |  |  |
|  | | AD | | 2.69 (2.41, 2.98) | | | 1.70 (1.51, 1.87) | | 1.70 (1.51, 1.87) | | | 1.70 (1.51, 1.87) | | |  | | |  |  |
|  | |  | |  | | |  | |  | | |  | | |  | | |  |  |
|  | | RD | | 0.80 (0.66, 0.91) | | | 0.75 (0.62, 0.86) | | 0.76 (0.61, 0.90) | | | 0.73 (0.61, 0.85) | | |  | | |  |  |
|  | |  | |  | | |  | |  | | |  | | |  | | |  |  |
| **healthy subjects** | | median nerve (upper arm) | | | | |  | |  | | |  | | |  | | |  |  |
|  | | FA | | 0.68 (0.63, 0.73) | | | 0.63 (0.58, 0.69) | | 0.63 (0.56, 0.68) | | | 0.63 (0.57, 0.69) | | |  | | |  |  |
|  | |  | |  | | |  | |  | | |  | | |  | | |  |  |
|  | | ADC | | 1.11 (1.02, 1.19) | | | 0.90 (0.83, 0.98) | | 0.90 (0.83, 0.98) | | | 0.90 (0.81, 0.97) | | |  | | |  |  |
|  | |  | |  | | |  | |  | | |  | | |  | | |  |  |
|  | | AD | | 2.32 (2.07, 2.71) | | | 1.64 (1.52, 1.79) | | 1.64 (1.52, 1.79) | | | 1.64 (1.52, 1.79) | | |  | | |  |  |
|  | |  | |  | | |  | |  | | |  | | |  | | |  |  |
|  | | RD | | 0.54 (0.47, 0.62) | | | 0.53 (0.46, 0.59) | | 0.53 (0.46, 0.60) | | | 0.52 (0.43, 0.60) | | |  | | |  |  |
|  | |  | |  | | |  | |  | | |  | | |  | | |  |  |
| **healthy subjects** | | radial nerve (upper arm) | | | | |  | |  | | |  | | |  | | |  |  |
|  | | FA | | 0.71 (0.65, 0.76) | | | 0.67 (0.62, 0.74) | | 0.66 (0.60, 0.73) | | | 0.70 (0.64, 0.74) | | |  | | |  |  |
|  | |  | |  | | |  | |  | | |  | | |  | | |  |  |
|  | | ADC | | 1.20 (1.10, 1.30) | | | 0.98 (0.91, 1.06) | | 1.01 (0.91, 1.09) | | | 0.97 (0.88, 1.05) | | |  | | |  |  |
|  | |  | |  | | |  | |  | | |  | | |  | | |  |  |
|  | | AD | | 2.70 (2.34, 3.07) | | | 1.89 (1.71, 2.10) | | 1.89 (1.71, 2.10) | | | 1.89 (1.71, 2.10) | | |  | | |  |  |
|  | |  | |  | | |  | |  | | |  | | |  | | |  |  |
|  | | RD | | 0.58 (0.45, 0.69) | | | 0.52 (0.46, 0.59) | | 0.54 (0.46, 0.62) | | | 0.50 (0.41, 0.56) | | |  | | |  |  |
|  | |  | |  | | |  | |  | | |  | | |  | | |  |  |
| **healthy subjects** | | ulnar nerve (upper arm) | | | | |  | |  | | |  | | |  | | |  |  |
|  | | FA | | 0.58 (0.53, 0.63) | | | 0.45 (0.36, 0.53) | | 0.44 (0.33, 0.53) | | | 0.46 (0.36, 0.54) | | |  | | |  |  |
|  | |  | |  | | |  | |  | | |  | | |  | | |  |  |
|  | | ADC | | 1.15 (1.02, 1.23) | | | 0.93 (0.79, 1.02) | | 0.94 (0.84, 1.03) | | | 0.92 (0.81, 1.04) | | |  | | |  |  |
|  | |  | |  | | |  | |  | | |  | | |  | | |  |  |
|  | | AD | | 2.13 (1.84, 2.44) | | | 1.44 (1.26, 1.59) | | 1.44 (1.26, 1.59) | | | 1.44 (1.26, 1.59) | | |  | | |  |  |
|  | |  | |  | | |  | |  | | |  | | |  | | |  |  |
|  | | RD | | 0.69 (0.55, 0.79) | | | 0.67 (0.56, 0.78) | | 0.68 (0.56, 0.79) | | | 0.64 (0.54, 0.78) | | |  | | |  |  |
|  | |  | |  | | |  | |  | | |  | | |  | | |  |  |
| **Suppl. Table 1: Values of fractional anisotropy (FA), apparent diffusion coefficient (ADC), axial diffusivity (AD), and radial diffusivity (RD).** Values are median (IQR). ADC [mm^2^/s], AD [10^-3^ mm^2^/s], RD [10^-3^ mm^2^/s]. | | | | | | | | | | | | | | | | |  | | |
|  | | | | | | | | | | |  | | |  | | |  | | |
|  |  | | | |  | | |  | | |  | | |  | | |  | | |

**Supplementary Table 2**

|  |  |  |  |  |  |  |  |  |  |  |  |  |  |  |
| --- | --- | --- | --- | --- | --- | --- | --- | --- | --- | --- | --- | --- | --- | --- |
|  |  | **DTI_20_** | **Model 1** | **Model 2** | **Model 3** |  |  |  |  |  |  |  |  |  |
|  |  |  |  |  |  |  |  |  |  |  |  |  |  |  |
| **healthy subjects** | tibial nerve (distal thigh) | |  |  |  |  |  |  |  |  |  |  |  |  |
|  | FA | 0.036 (0.024, 0.063) | 0.048 (0.029, 0.079) | 0.057 (0.033, 0.103) | 0.056 (0.029, 0.091) |  |  |  |  |  |  |  |  |  |
|  |  |  |  |  |  |  |  |  |  |  |  |  |  |  |
|  | ADC | 0.074 (0.041, 0.094) | 0.081 (0.062, 0.116) | 0.086 (0.062, 0.140) | 0.080 (0.065, 0.104) |  |  |  |  |  |  |  |  |  |
|  |  |  |  |  |  |  |  |  |  |  |  |  |  |  |
|  | AD | 0.200 (0.108, 0.367) | 0.166 (0.126, 0.211) | 0.166 (0.126, 0.211) | 0.166 (0.126, 0.211) |  |  |  |  |  |  |  |  |  |
|  |  |  |  |  |  |  |  |  |  |  |  |  |  |  |
|  | RD | 0.067 (0.051, 0.120) | 0.063 (0.043, 0.110) | 0.078 (0.051, 0.160) | 0.067 (0.055, 0.090) |  |  |  |  |  |  |  |  |  |
|  |  |  |  |  |  |  |  |  |  |  |  |  |  |  |
| **age-matched controls** | tibial nerve (distal thigh) | |  |  |  |  |  |  |  |  |  |  |  |  |
|  | FA | 0.039 (0.029, 0.050) | 0.058 (0.039, 0.102) | 0.083 (0.051, 0.130) | 0.066 (0.035, 0.116) |  |  |  |  |  |  |  |  |  |
|  |  |  |  |  |  |  |  |  |  |  |  |  |  |  |
|  | ADC | 0.085 (0.048, 0.189) | 0.094 (0.071, 0.170) | 0.102 (0.069, 0.151) | 0.104 (0.079, 0.195) |  |  |  |  |  |  |  |  |  |
|  |  |  |  |  |  |  |  |  |  |  |  |  |  |  |
|  | AD | 0.219 (0.107, 0.330) | 0.157 (0.116, 0.215) | 0.157 (0.116, 0.215) | 0.157 (0.116, 0.215) |  |  |  |  |  |  |  |  |  |
|  |  |  |  |  |  |  |  |  |  |  |  |  |  |  |
|  | RD | 0.077 (0.036, 0.189) | 0.094 (0.058, 0.162) | 0.119 (0.067, 0.143) | 0.105 (0.064, 0.200) |  |  |  |  |  |  |  |  |  |
|  |  |  |  |  |  |  |  |  |  |  |  |  |  |  |
| **patients** | tibial nerve (distal thigh) | |  |  |  |  |  |  |  |  |  |  |  |  |
|  | FA | 0.041 (0.020, 0.057) | 0.054 (0.028, 0.078) | 0.065 (0.041, 0.100) | 0.062 (0.031, 0.089) |  |  |  |  |  |  |  |  |  |
|  |  |  |  |  |  |  |  |  |  |  |  |  |  |  |
|  | ADC | 0.086 (0.035, 0.135) | 0.089 (0.054, 0.149) | 0.103 (0.061, 0.156) | 0.102 (0.059, 0.165) |  |  |  |  |  |  |  |  |  |
|  |  |  |  |  |  |  |  |  |  |  |  |  |  |  |
|  | AD | 0.28  (0.157, 0.453) | 0.140  (0.094, 0.209) | 0.140 (0.094, 0.209) | 0.140 (0.094, 0.209) |  |  |  |  |  |  |  |  |  |
|  |  |  |  |  |  |  |  |  |  |  |  |  |  |  |
|  | RD | 0.073 (0.044, 0.146) | 0.085 (0.059, 0.174) | 0.118 (0.072, 0.191) | 0.091 (0.059, 0.192) |  |  |  |  |  |  |  |  |  |
|  |  |  |  |  |  |  |  |  |  |  |  |  |  |  |
| **healthy subjects** | tibial nerve (prox. calf) | |  |  |  |  |  |  |  |  |  |  |  |  |
|  | FA | 0.057 (0.034, 0.084) | 0.071 (0.044, 0.085) | 0.084 (0.054, 0.109) | 0.081 (0.052, 0.111) |  |  |  |  |  |  |  |  |  |
|  |  |  |  |  |  |  |  |  |  |  |  |  |  |  |
|  | ADC | 0.133 (0.067, 0.195) | 0.147 (0.102, 0.223) | 0.142 (0.088, 0.243) | 0.150 (0.115, 0.208) |  |  |  |  |  |  |  |  |  |
|  |  |  |  |  |  |  |  |  |  |  |  |  |  |  |
|  | AD | 0.375 (0.207, 0.801) | 0.239 (0.182, 0.296) | 0.239 (0.182, 0.296) | 0.239 (0.182, 0.296) |  |  |  |  |  |  |  |  |  |
|  |  |  |  |  |  |  |  |  |  |  |  |  |  |  |
|  | RD | 0.147 (0.112, 0.188) | 0.122 (0.088, 0.209) | 0.126 (0.068, 0.248) | 0.129 (0.120, 0.179) |  |  |  |  |  |  |  |  |  |
|  |  |  |  |  |  |  |  |  |  |  |  |  |  |  |
| **healthy subjects** | median nerve (upper arm) | |  |  |  |  |  |  |  |  |  |  |  |  |
|  | FA | 0.042 (0.028, 0.068) | 0.051 (0.036, 0.074) | 0.072 (0.041, 0.104) | 0.056 (0.039, 0.094) |  |  |  |  |  |  |  |  |  |
|  |  |  |  |  |  |  |  |  |  |  |  |  |  |  |
|  | ADC | 0.080 (0.052, 0.114) | 0.084 (0.065, 0.107) | 0.087 (0.072, 0.117) | 0.087  (0.056, 0.117) |  |  |  |  |  |  |  |  |  |
|  |  |  |  |  |  |  |  |  |  |  |  |  |  |  |
|  | AD | 0.322 (0.110, 0.747) | 0.170 (0.111, 0.241) | 0.170 (0.111, 0.241) | 0.170 (0.111, 0.241) |  |  |  |  |  |  |  |  |  |
|  |  |  |  |  |  |  |  |  |  |  |  |  |  |  |
|  | RD | 0.080 (0.057, 0.133) | 0.068 (0.046, 0.096) | 0.086 (0.047, 0.151) | 0.075 (0.047, 0.106) |  |  |  |  |  |  |  |  |  |
|  |  |  |  |  |  |  |  |  |  |  |  |  |  |  |
| **healthy subjects** | radial nerve (upper arm) | |  |  |  |  |  |  |  |  |  |  |  |  |
|  | FA | 0.054 (0.035, 0.094) | 0.063 (0.038, 0.123) | 0.075 (0.043, 0.188) | 0.065 (0.039, 0.090) |  |  |  |  |  |  |  |  |  |
|  |  |  |  |  |  |  |  |  |  |  |  |  |  |  |
|  | ADC | 0.092 (0.057, 0.168) | 0.105 (0.061, 0.179) | 0.109 (0.066, 0.221) | 0.099 (0.060, 0.145) |  |  |  |  |  |  |  |  |  |
|  |  |  |  |  |  |  |  |  |  |  |  |  |  |  |
|  | AD | 0.419 (0.293, 0.762) | 0.229 (0.151, 0.275) | 0.229 (0.151, 0.275) | 0.229 (0.151, 0.275) |  |  |  |  |  |  |  |  |  |
|  |  |  |  |  |  |  |  |  |  |  |  |  |  |  |
|  | RD | 0.111 (0.071, 0.135) | 0.088 (0.040, 0.177) | 0.121 (0.051, 0.249) | 0.081 (0.041, 0.118) |  |  |  |  |  |  |  |  |  |
|  |  |  |  |  |  |  |  |  |  |  |  |  |  |  |
| **healthy subjects** | ulnar nerve (upper arm) | |  |  |  |  |  |  |  |  |  |  |  |  |
|  | FA | 0.054 (0.037, 0.093) | 0.091 (0.062, 0.120) | 0.112 (0.071, 0.158) | 0.095 (0.063, 0.136) |  |  |  |  |  |  |  |  |  |
|  |  |  |  |  |  |  |  |  |  |  |  |  |  |  |
|  | ADC | 0.114 (0.045, 0.172) | 0.107 (0.068, 0.182) | 0.102 (0.075, 0.138) | 0.110 (0.077, 0.157) |  |  |  |  |  |  |  |  |  |
|  |  |  |  |  |  |  |  |  |  |  |  |  |  |  |
|  | AD | 0.368 (0.152, 0.524) | 0.175 (0.087, 0.272) | 0.175 (0.087, 0.272) | 0.175 (0.087, 0.272) |  |  |  |  |  |  |  |  |  |
|  |  |  |  |  |  |  |  |  |  |  |  |  |  |  |
|  | RD | 0.106 (0.078, 0.178) | 0.102 (0.082, 0.141) | 0.131 (0.087, 0.162) | 0.126 (0.082, 0.167) |  |  |  |  |  |  |  |  |  |
|  |  |  |  |  |  |  |  |  |  |  |  |  |  |  |
| **Suppl. Table 2: Standard deviation (SD) of fractional anisotropy (FA), apparent diffusion coefficient (ADC), axial diffusivity (AD), and radial diffusivity (RD) values.** Values are median (min-max). | | | | | | |  |  |  |  |  |  |  |  |

**Supplementary Table 3**

|  |  | **Angulation [°]** | **Intraneurial  connective tissue [%]** |
| --- | --- | --- | --- |
|  |  |  |  |
| **healthy subjects** | tibial nerve (distal thigh) | 10.00 (1.28, 30.30) | 1.04 (0.00, 26.67) |
|  |  |  |  |
| **age-matched controls** | tibial nerve (distal thigh) | 12.00 (1.82, 24.00) | 11.89 (0.00, 46.84) |
|  |  |  |  |
| **patients** | tibial nerve (distal thigh) | 12.40 (0.97, 35.20) | 6.49 (0.00, 57.50) |
|  |  |  |  |
| **healthy subjects** | tibial nerve (proximal calf) | 9.68 (0.96, 28.70) | n.a. |
|  |  |  |  |
| **healthy subjects** | median nerve (upper arm) | 5.79 (0.31, 15.80) | n.a. |
|  |  |  |  |
| **healthy subjects** | radial nerve (upper arm) | 13.30 (2.22, 30.50) | n.a. |
|  |  |  |  |
| **healthy subjects** | ulnar nerve (upper arm) | 8.97 (0.73, 23.30) | n.a. |
|  |  |  |  |
| **Suppl. Table 3: Values of nerve angulation and intraneurial connective tissue across regions.** Values are median (min-max). | | | |

**Supplementary Table 4**

|  |  | **DTI_20_** | | **Model 1** | | **Model 2** | | **Model 3** | |
| --- | --- | --- | --- | --- | --- | --- | --- | --- | --- |
|  |  | ***r*** | ***p*** | ***r*** | ***p*** | ***r*** | ***p*** | ***r*** | ***p*** |
| **healthy subjects** | tibial nerve  (distal thigh) | -0.49 | **<.001** | -0.38 | **<.001** | -0.32 | **<.001** | -0.41 | **<.001** |
|  |  |  |  |  |  |  |  |  |  |
| **age-matched controls** | tibial nerve  (distal thigh) | -0.30 | **<.001** | -0.14 | **0.040** | -0.12 | 0.082 | -0.13 | 0.066 |
|  |  |  |  |  |  |  |  |  |  |
| **patients** | tibial nerve  (distal thigh) | -0.18 | **0.010** | -0.12 | 0.078 | -0.17 | **0.013** | -0.06 | 0.376 |
|  |  |  |  |  |  |  |  |  |  |
| **Suppl. Table 4a: Correlation analysis of absolute fractional anisotropy values and intraneurial connective tissue.** P values <.05 are marked in bold letters. r, Spearman's correlation coefficient. | | | | | | | | | |
|  | | | |  |  |  |  |  |  |
|  |  |  |  |  |  |  |  |  |  |
|  |  | **Model 1** | | **Model 2** | | **Model 3** | |  | |
|  |  | ***r*** | ***p*** | ***r*** | ***p*** | ***r*** | ***p*** |  |  |
| **healthy subjects** | tibial nerve (distal thigh) | -0.08 | 0.313 | -0.14 | 0.068 | 0.01 | 0.946 |  |  |
|  |  |  |  |  |  |  |  |  |  |
| **age-matched  controls** | tibial nerve (distal thigh) | -0.11 | 0.098 | -0.07 | 0.302 | -0.08 | 0.264 |  |  |
|  |  |  |  |  |  |  |  |  |  |
| **patients** | tibial nerve (distal thigh) | -0.02 | 0.785 | 0.10 | 0.160 | -0.11 | 0.117 |  |  |
|  |  |  |  |  |  |  |  |  |  |
| **Suppl. Table 4b: Correlation analysis of accuracy of fractional anisotropy values and intraneurial connective tissue.** P values <.05 are marked in bold letters. r, Spearman's correlation coefficient. | | | | | | | |  |  |
|  | | | |  |  |  |  |  |  |

**Supplementary Table 5**

|  |  | **DTI_20_** | | **Model 1** | | **Model 2** | | **Model 3** | |
| --- | --- | --- | --- | --- | --- | --- | --- | --- | --- |
|  |  | ***r*** | ***p*** | ***r*** | ***p*** | ***r*** | ***p*** | ***r*** | ***p*** |
| **healthy subjects** | tibial nerve (distal thigh) | 0.02 | 0.778 | -0.08 | 0.291 | -0.04 | 0.584 | -0.13 | 0.106 |
|  |  |  |  |  |  |  |  |  |  |
| **age-matched  controls** | tibial nerve (distal thigh) | 0.15 | **0.043** | -0.11 | 0.135 | -0.07 | 0.313 | -0.11 | 0.140 |
|  |  |  |  |  |  |  |  |  |  |
| **patients** | tibial nerve (distal thigh) | -0.05 | 0.485 | -0.15 | **0.037** | -0.09 | 0.215 | -0.20 | **0.005** |
|  |  |  |  |  |  |  |  |  |  |
| **healthy subjects** | tibial nerve (prox. calf) | 0.14 | 0.079 | 0.16 | **0.049** | 0.14 | 0.071 | 0.13 | 0.105 |
|  |  |  |  |  |  |  |  |  |  |
| **healthy subjects** | median nerve (upper arm) | 0.30 | <.001 | 0.23 | **0.004** | 0.11 | 0.169 | 0.30 | **<.001** |
|  |  |  |  |  |  |  |  |  |  |
| **healthy subjects** | radial nerve (upper arm) | 0.20 | **0.012** | -0.05 | 0.561 | -0.16 | **0.042** | 0.11 | 0.175 |
|  |  |  |  |  |  |  |  |  |  |
| **healthy subjects** | ulnar nerve (upper arm) | 0.10 | 0.232 | -0.29 | **<.001** | -0.28 | **<.001** | -0.25 | **0.001** |
|  |  |  |  |  |  |  |  |  |  |
| **Suppl. Table 5a: Correlation analysis of absolute fractional anisotropy values and nerve angulation.** P values <.05 are marked in bold letters. r, Spearman's correlation coefficient. | | | | | | | | | |
|  |  | **Model 1** | | **Model 2** | | **Model 3** | |  | |
|  |  | ***r*** | ***p*** | ***r*** | ***p*** | ***r*** | ***p*** |  |  |
| **healthy subjects** | tibial nerve (distal thigh) | 0.23 | **0.003** | 0.14 | 0.086 | 0.17 | **0.031** |  |  |
|  |  |  |  |  |  |  |  |  |  |
| **age-matched  controls** | tibial nerve (distal thigh) | 0.25 | **<.001** | 0.17 | **0.015** | 0.21 | **0.004** |  |  |
|  |  |  |  |  |  |  |  |  |  |
| **patients** | tibial nerve (distal thigh) | 0.23 | **0.001** | 0.13 | 0.071 | 0.25 | **<.001** |  |  |
|  |  |  |  |  |  |  |  |  |  |
| **healthy subjects** | tibial nerve (proximal calf) | 0.06 | 0.476 | -0.02 | 0.825 | 0.09 | 0.271 |  |  |
|  |  |  |  |  |  |  |  |  |  |
| **healthy subjects** | median nerve (upper arm) | -0.02 | 0.808 | 0.06 | 0.464 | -0.14 | 0.070 |  |  |
|  |  |  |  |  |  |  |  |  |  |
| **healthy subjects** | radial nerve (upper arm) | 0.19 | **0.015** | 0.35 | **<.001** | -0.06 | 0.478 |  |  |
|  |  |  |  |  |  |  |  |  |  |
| **healthy subjects** | ulnar nerve (upper arm) | 0.35 | **<.001** | 0.29 | **<.001** | 0.31 | **<.001** |  |  |
|  |  |  |  |  |  |  |  |  |  |
| **Suppl. Table 5b: Correlation analysis of accuracy of fractional anisotropy values and nerve angulation.** P values <.05 are marked in bold letters. r, Spearman's correlation coefficient. | | | | | | | | |  |

**Supplementary Figures**


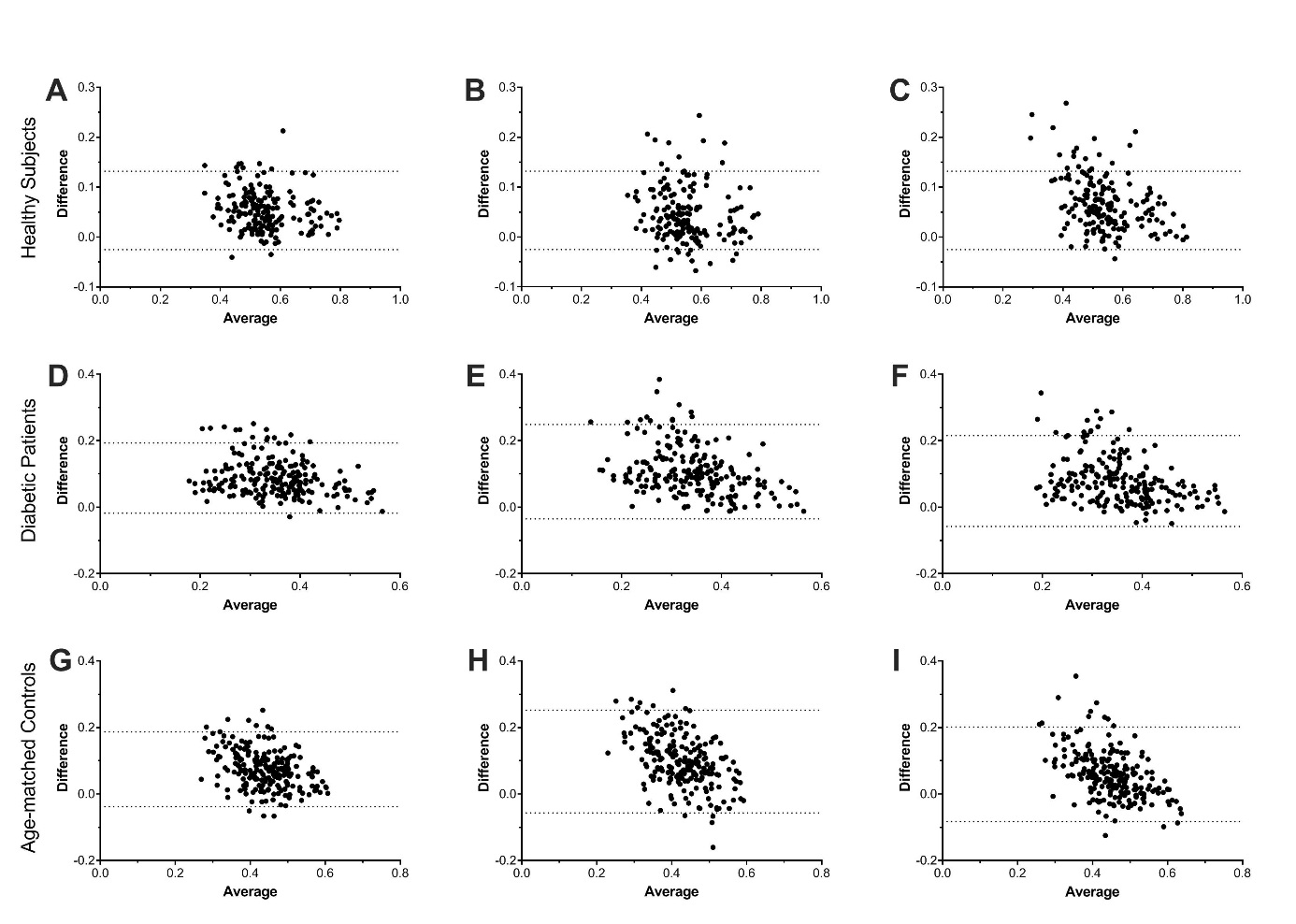


**Supplementary Figure 1:** **Accuracy of FA as assessed by Bland-Altman plots**

Bland-Altman plots showing the limits of difference (± 2 SD) in healthy subjects (upper panel), diabetic patients (middle panel) and age-matched controls (lower panel) between paired values of fractional anisotropy (FA) calculated with 20-vector DTI and model 1 (A, D, G), model 2 (B, E, H), or model 3 (C, F, I) in the tibial nerve at thigh level.


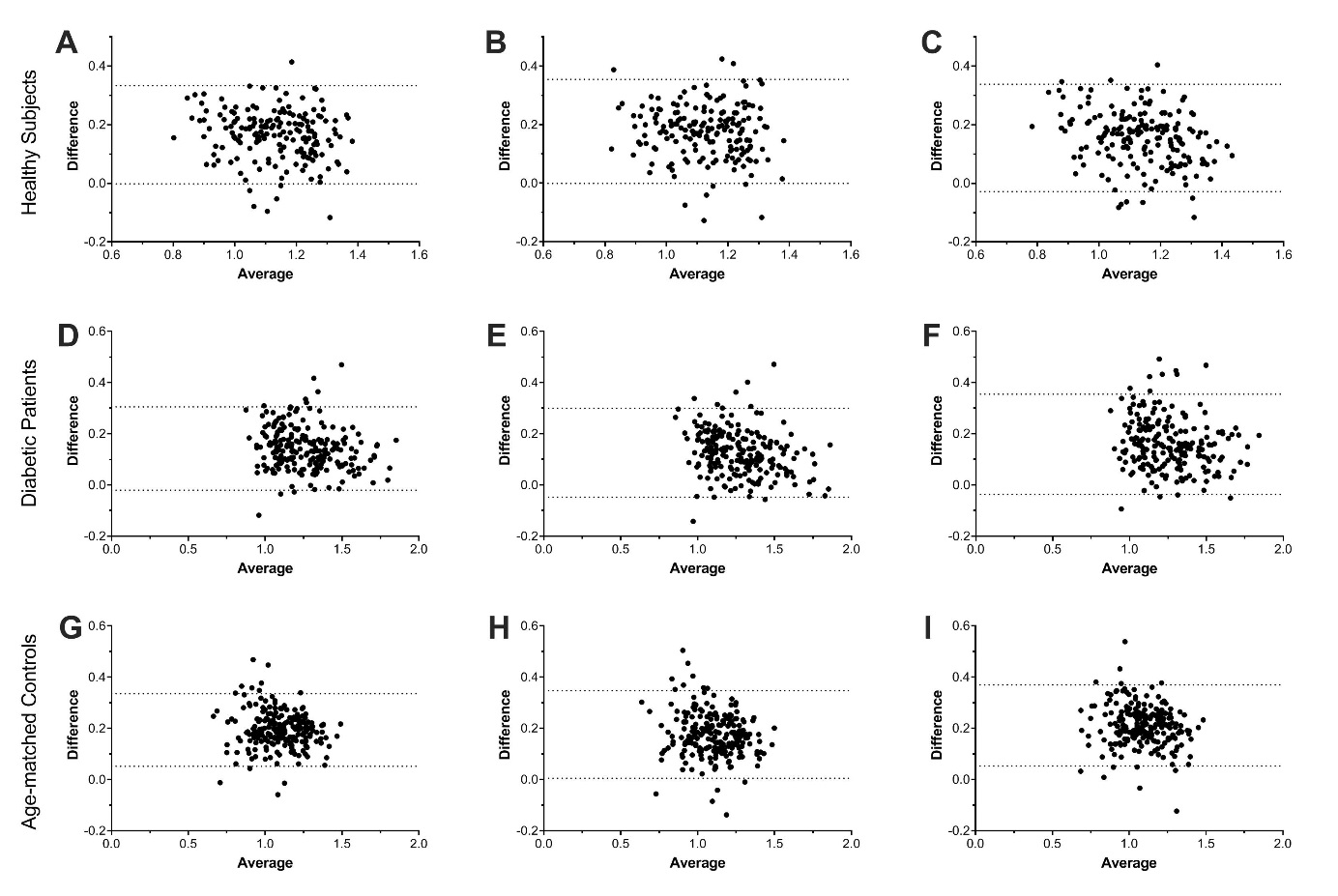


**Supplementary Figure 2:** **Accuracy of ADC as assessed by Bland-Altman plots**

Bland-Altman plots showing the limits of difference (± 2 SD) in healthy subjects (upper panel), diabetic patients (middle panel) and age-matched controls (lower panel) between paired values of apparent diffusion coefficient (ADC) calculated with 20-vector DTI and model 1 (A, D, G), model 2 (B, E, H), or model 3 (C, F, I) in the tibial nerve at thigh level.


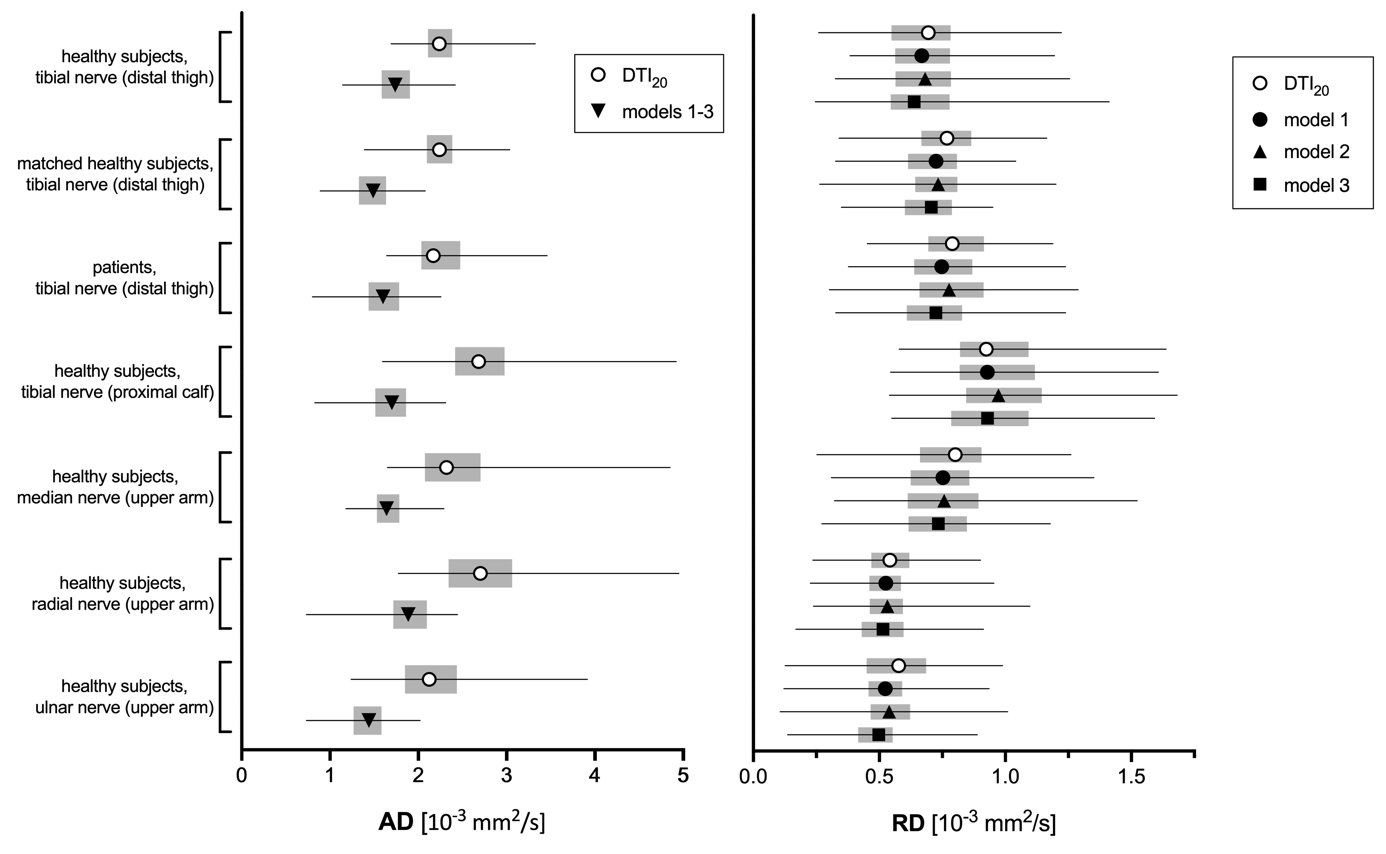


**Supplementary Figure 3:** **Accuracy of different AD and RD calculation models**

Forest plots of absolute axial diffusivity (AD, left side) and radial diffusivity (right side) show the accuracy of models 1-3 compared to the 20-directional standard model (DTI_20_). Symbols indicate the median, horizontal lines depict the range, and gray boxes show the interquartile range.

**Supplementary Figure 4:** **Precision of different AD and RD calculation models**

Forest plots of within-subject precision of axial diffusivity (AD, left side) and radial diffusivity (RD, right side) show the precision of models 1-3 compared to the 20-directional standard model (DTI_20_). Standard deviation is plotted on the x-axes. Symbols indicate the median, horizontal lines depict the range, and gray boxes show the interquartile range. Significant differences between models 1-3 to DTI_20_ are indicated with asterisks (* p<.05; ** p<.01, ** p<.001).


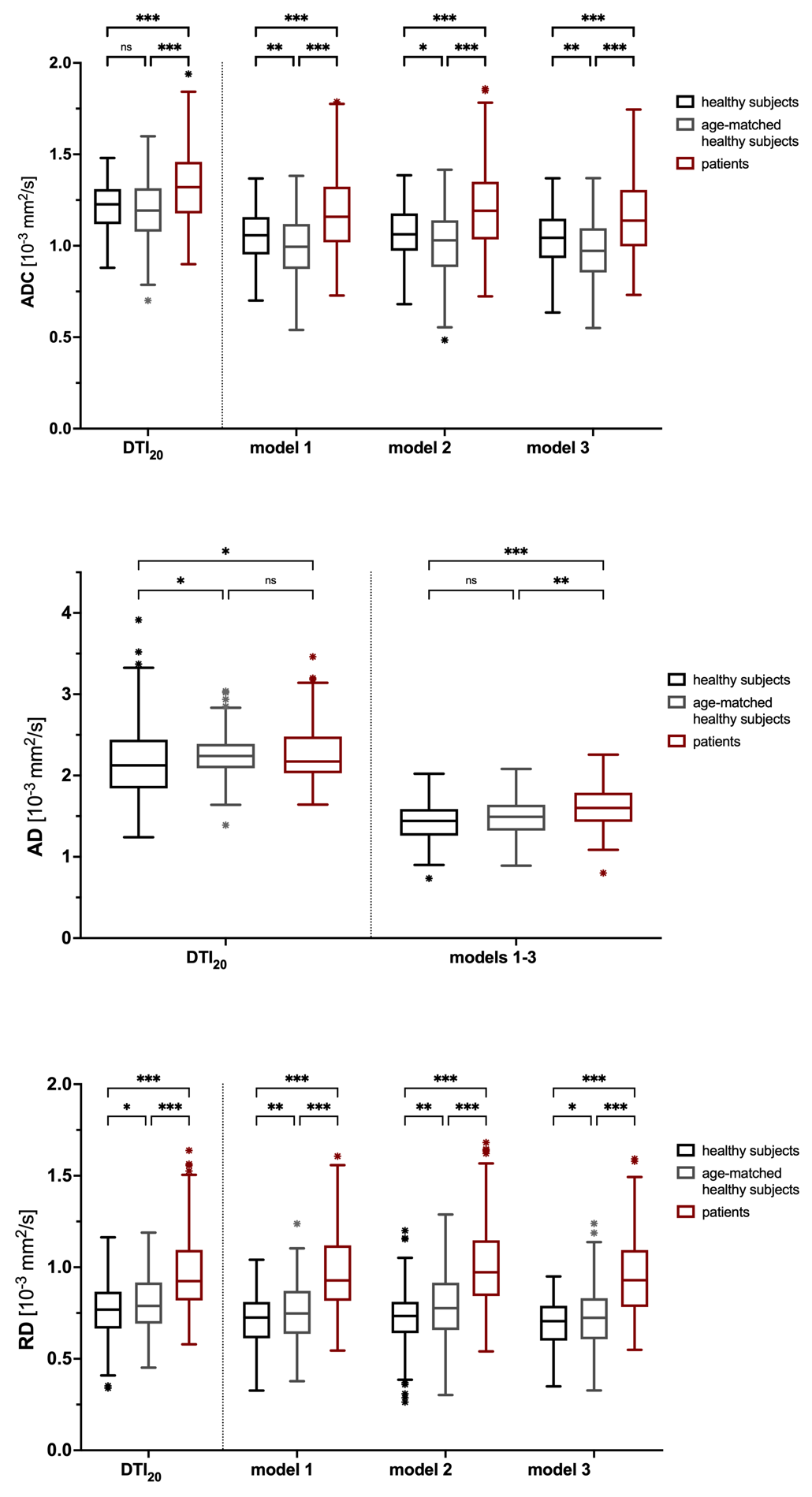


**Supplementary Figure 5:** **Group differences based on different ADC, AD and RD calculation models**

Box-and-whisker plots of apparent diffusion coefficient (ADC) and axial and radial diffusivity (AD, RD) measured in the tibial nerve at the distal thigh show group differences between diabetic patients and healthy subjects using the four analyzed models. Data are medians (lines in boxes), 25^th^ to 75^th^ percentiles (bottom and top of boxes), and ranges (Tukey whiskers).


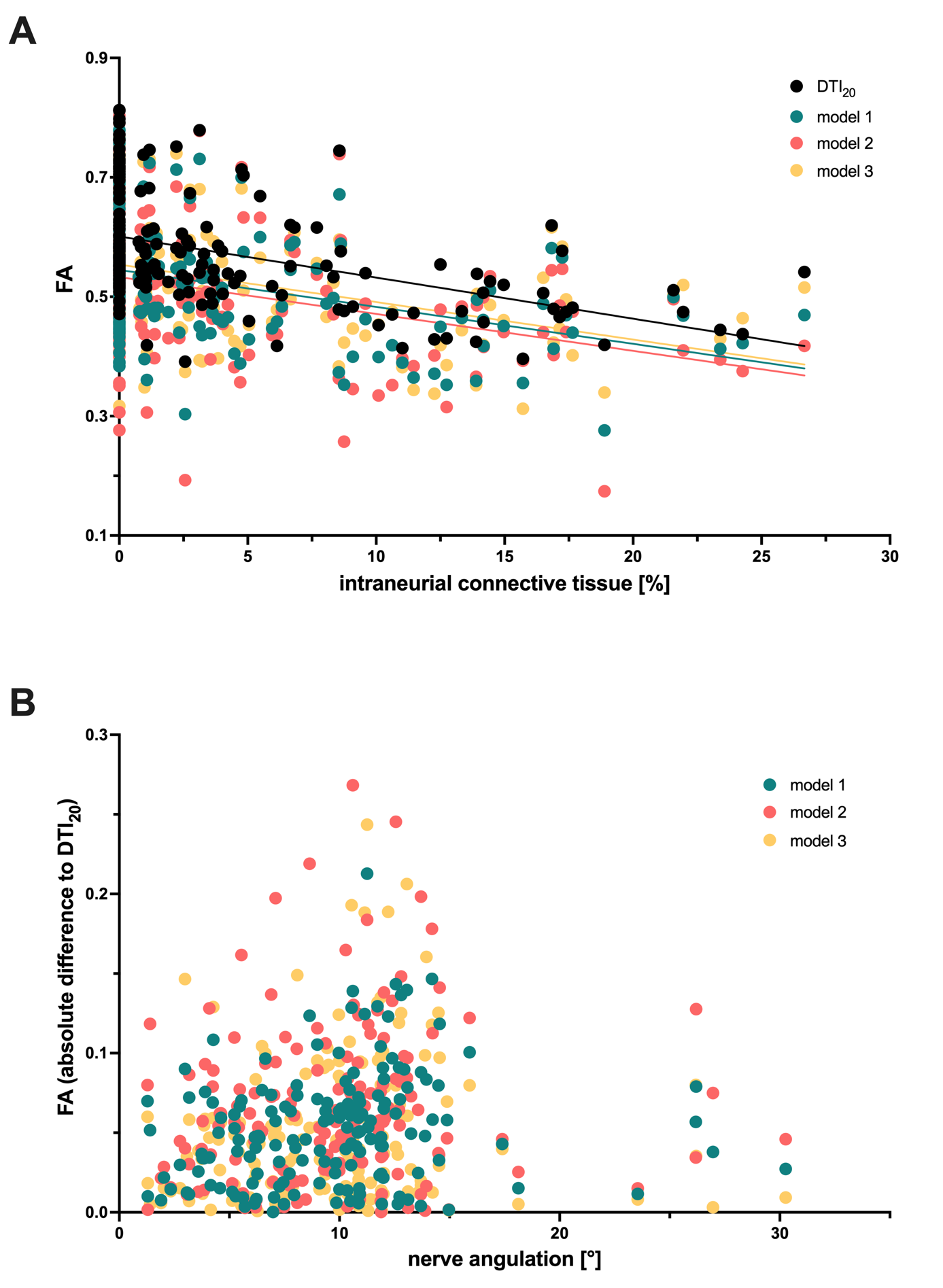


**Supplementary Figure 6:** **FA dependence on intraneurial connective tissue and nerve angulation**

Scatter plot of fractional anisotropy (FA) measured in the tibial nerve at the distal thigh in young healthy subjects in relation to percentage of intraneurial connective tissue shows a negative correlation in all four examined models (A), while no clear dependence was detected between FA accuracy and nerve angulation (B).
